# Radio-Frequency-Based NH_3_-Selective Catalytic Reduction Catalyst Control: Studies on Temperature Dependency and Humidity Influences

**DOI:** 10.3390/s17071615

**Published:** 2017-07-12

**Authors:** Markus Dietrich, Gunter Hagen, Willibald Reitmeier, Katharina Burger, Markus Hien, Philippe Grass, David Kubinski, Jaco Visser, Ralf Moos

**Affiliations:** 1 Bayreuth Engine Research Center (BERC), Department of Functional Materials, University of Bayreuth, 95447 Bayreuth, Germany; Markus.Dietrich@continental-corporation.com (M.D.); functional.materials@uni-bayreuth.de (G.H.); 2 Continental Automotive GmbH, Division Powertrain, Siemensstraße 12, 93055 Regensburg, Germany; Willibald.Reitmeier@continental-corporation.com (W.R.); Katharina.2.Burger@continental-corporation.com (K.B.); Markus.Hien@continental-corporation.com (M.H.); Philippe.Grass@continental-corporation.com (P.G.); 3 Ford Research and Innovation Center, 2101 Village Rd., Dearborn, MI 48124, USA; dkubinsk@ford.com (D.K.); jvisser@ford.com (J.V.)

**Keywords:** radio-frequency (RF), NH_3_ SCR, NH_3_ storage, direct control, microwave cavity perturbation, exhaust gas sensor, cold start

## Abstract

The upcoming more stringent automotive emission legislations and current developments have promoted new technologies for more precise and reliable catalyst control. For this purpose, radio-frequency-based (RF) catalyst state determination offers the only approach for directly measuring the NH_3_ loading on selective catalytic reduction (SCR) catalysts and the state of other catalysts and filter systems. Recently, the ability of this technique to directly control the urea dosing on a current NH_3_ storing zeolite catalyst has been demonstrated on an engine dynamometer for the first time and this paper continues that work. Therefore, a well-known serial-type and zeolite-based SCR catalyst (Cu-SSZ-13) was investigated under deliberately chosen high space velocities. At first, the full functionality of the RF system with Cu-SSZ-13 as sample was tested successfully. By direct RF-based NH_3_ storage control, the influence of the storage degree on the catalyst performance, i.e., on NO_x_ conversion and NH_3_ slip, was investigated in a temperature range between 250 and 400 °C. For each operation point, an ideal and a critical NH_3_ storage degree was found and analyzed in the whole temperature range. Based on the data of all experimental runs, temperature dependent calibration functions were developed as a basis for upcoming tests under transient conditions. Additionally, the influence of exhaust humidity was observed with special focus on cold start water and its effects to the RF signals.

## 1. Introduction

Continuously tightening vehicle emission legislations are the main driving factor for improvements in engine and exhaust gas aftertreatment technologies among automotive manufacturers worldwide [1]. Especially diesel engine driven vehicles and their higher emission of nitric oxides (NO_x_ = NO + NO_2_) are in focus of research, development and the media [2,3]. The selective catalytic reduction (SCR) using ammonia (NH_3_) as reducing agent is today’s main deNO_x_ technology for light and heavy duty diesel engines. In this technology, an aqueous solution of 32.5 wt % urea in water (diesel exhaust fluid = DEF, AdBlue^TM^ or AUS32 = aqueous urea solution) is injected into the exhaust and decomposes after water evaporation by thermolysis and hydrolysis into gaseous NH_3_ and carbon dioxide (CO_2_). The formed NH_3_ adsorbs on the active sites of the SCR catalyst and can react with NO_x_ to form nitrogen (N_2_) and water (H_2_O) [2,4]. Relying on current schemes of the SCR reaction mechanism for several SCR catalysts, the prior NH_3_ adsorption is an essential precondition for all SCR reactions [5,6,7,8]. Depending on the NO/NO_2_ ratio, different SCR reactions occur on the catalyst surface. The two main reactions are the standard SCR reaction (Equation (1)) only with NO and oxygen (O_2_), and the fast SCR reaction with equimolar amounts of NO and NO_2_ without participation of O_2_ (Equation (2)) [2,4]:

4NH_3_ + 4NO + O_2_ → 4N_2_ + 6 H_2_O (1)

4NH_3_ + 2NO + 2NO_2_ → 4N_2_ + 6H_2_O (2)

Beside its necessity for the SCR reactions, the prior NH_3_ adsorption and storage on the catalyst is also beneficial in application to changing concentration and flow conditions in the buffer related to transient driving. Due to the kinetic limitations of the SCR reactions, a sufficient NH_3_ surface coverage is also required to achieve good NO_x_ conversion efficiencies [5,9]. Additionally, it is necessary to avoid too high storage degrees, since this may lead to NH_3_ slip. Consequently, the catalyst control is required to secure an NH_3_ storage degree always between the minimum storage for high conversion and the maximum storage without NH_3_ slip to meet the current emission limits [10,11]. Therefore, the development also aims for SCR catalyst materials with high NH_3_ storage capacity and high low-temperature activity, such as copper (Cu) exchanged zeolites [12,13,14,15].

The current DEF dosing control is completely model-based and relies on gas sensor signals, i.e., from NO_x_ and/or NH_3_ sensors [10]. In these approaches, the whole ad- and desorption equilibrium and all reactions occurring on the catalyst surface are simulated and the necessary amount of DEF is calculated [11,16,17]. This also requires injector self-diagnosis and urea concentration monitoring to secure the functionality of the whole SCR system. Small errors and deviations of only one part of this system may lead to incorrect urea dosing followed by NO_x_ or NH_3_ emissions [18,19]. A measurement system to determine the current NH_3_ loading on the catalyst for model validation or direct dosing control on the road is not yet available.

The radio-frequency-based (RF) catalyst and filter state determination technique has been a focus of research and development for several years. Since it operates in the range between 1 and 3 GHz, it sometimes also denoted as microwave-based state determination. With the RF technique, a contactless and direct (*in-operando*) measure of the catalyst/filter states, by using the metal catalyst canning as an electrical cavity resonator was presented [20,21,22]. At first, the oxidation state of three-way catalytic converters (TWC) was determined, indicating that the RF approach is capable of providing more precise information about the catalyst state and its optimal operation point compared to established gas sensor- and model-based procedures [23,24,25]. The state of diesel or gasoline particulate filters (DPF or GPF) was also successfully monitored with the RF signal as a measure of accumulated soot [26,27,28,29,30]. First approaches to separate the signals of soot and ash appear promising [31]. Studies with a combined system of a TWC-coated GPF on an engine dynamometer proved the system functionality under transient conditions within the European driving cycle (NEDC) [30]. In NO_x_ reduction application, the storage state of lean NO_x_ traps (LNT) was successfully monitored, but application to this catalyst type seems to suffer from a comparably small signal [32,33]. The potential of the RF technique to determine the NH_3_ storage on SCR catalysts is presumed and is the focus of this paper. Previous work already proved the functionality for vanadia- and zeolite-based SCR catalysts [34,35,36,37]. Recently, we presented first results with a commercial zeolite-based catalyst on the engine dynamometer using for first time DEF instead of gaseous NH_3_. The next big step by applying a direct RF-controlled DEF dosing on a specific NH_3_ storage value was achieved [38]. This paper continues that work with a focus on the temperature dependence of the RF signal, the influence of the NH_3_ storage on NH_3_ slip and maximum NO_x_ conversion efficiency. The effect of humidity changes and the cold start behavior to the RF signal are also investigated. Within our work, we try to demonstrate possible benefits of a directly NH_3_ storage-controlled SCR catalyst to operate the whole SCR system at its optimal NO_x_ conversion point and to avoid NH_3_ slip. This might lead to increased system efficiency and more robust catalyst control systems for future applications and emission limits.

## 2. RF Catalyst State Monitoring

In the applied measurement technique, the catalyst itself is the sensitive part of the sensor system. By storing NH_3_, the catalyst material changes its electric properties and the cavity resonator, which is defined by the electrically conductive catalyst canning, is able to detect these very small changes. By coupling electromagnetic waves into the resonator, resonances, i.e., standing electromagnetic waves, can be excited at specific frequencies and their electric field interacts with the resonator filling. As a measurable material effect, the complex dielectric permittivity (*ε* = *ε*_1_ − j*ε_2_*) of the catalyst is identified. The linear relation between the NH_3_ loading and both parts of the complex permittivity for zeolite SCR catalyst materials has been proven in several studies with a special setup using powder samples [39,40,41]. The currently expected material effects due to NH_3_ adsorption are the polar nature of the NH_3_ molecule and the effects of NH_3_ to the conductivity mechanisms inside the porous zeolite structure [42,43].

Each resonance can be fully described by two analyzable resonance parameters: the resonance frequency *f*_res_ and the unloaded quality factor *Q*_0_. The absolute frequency of *f*_res_ is mainly defined by the resonance cavity geometry and the properties of the resonator filling material. Based on the theory of the so-called cavity perturbation method, small changes of the resonance frequency Δ*f*_res_/*f*_0_ depend on the changes of the dielectric permittivity Δ*ε*_1_, which represents the polarization effects (Equation (3)). Similarly, the changes of conductivity mechanisms and dielectric losses are represented in Δ*ε*_2_ and related to the change of the reciprocal unloaded quality factor Δ*Q*_0_^−1^ (Equation (4)):

Δ*f*_res_/*f*_0_ ∝ Δ*ε*_1_ (3)

Δ*Q*_0_^−1^ ∝ Δ*ε*_2_ (4)

Further detailed descriptions and the theoretical background of the RF measurement technique, including the used assumptions and the extraction of the two resonance parameters *f*_res_ and *Q*_0_ can be found in previous work [30,39,42].

It is possible to perform RF measurements with only one coupling element in simple reflection mode. By applying two coupling elements, the number of possible RF signals increases to four with two reflection and two transmission signals. Within this work, two coaxial probe antennas were used as coupling elements and the RF analysis is based on one transmission signal, the scattering parameter *S*_21_. By acquiring complex RF data, the data analysis uses a complex fitting approach for *f*_res_ and *Q*_0_ determination.

## 3. Experimental

**Figure 1:**
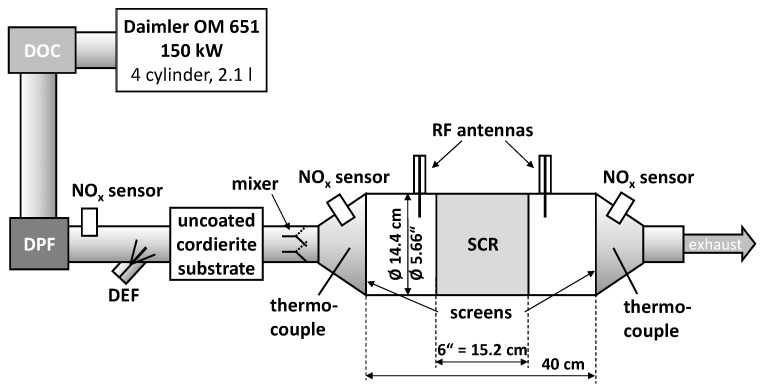
Illustration of the dynamometer setup: 2.1 l diesel engine with diesel oxidation catalyst and particulate filter, DEF dosing with uncoated cordierite substrate and plate mixer, Ø 5.66” (Ø 14.2 cm) SCR catalyst canning defined by metal screens with two RF antennas, thermocouples up- and downstream of the SCR and three NO_x_ sensors up- and downstream of SCR and upstream of DEF dosing.

**Figure 2:**
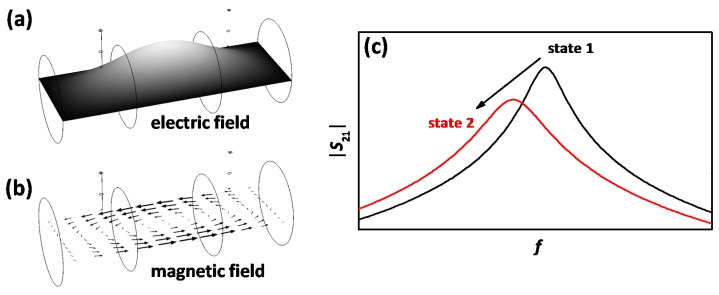
Simulated (**a**) electric field strength (high: light, low: black) and (**b**) magnetic field vectors of the TE_111_ mode; and (**c**) example transmission spectrum of the TE_111_ mode without NH_3_ (state 1, black) and loaded with NH_3_ (state 2, red).

The presented study uses the same dynamometer setup as described in [38]. Under investigation is a well-studied serial-type copper-exchanged zeolite SCR catalyst (Cu-SSZ-13 [36,37,44], kindly provided by the Ford Motor Company) on a cordierite substrate. The illustrated setup in Figure 1 is described as follows: a turbocharged 4-cylinder and 2.1 l diesel engine (Daimler OM 651, 150 kW) is followed by the serial device oxidation catalyst (DOC) and DPF. The first located NO_x_ sensor detects the pre-SCR NO_x_ raw emissions. The DEF dosing (Bosch Denoxtronic 3.2) is applied together with an uncoated cordierite substrate to support NH_3_ formation from the DEF with additional surface contact and a plate mixer to improve NH_3_ concentration uniformity. The second NO_x_ sensor determines together with the first one and its well-known NH_3_ cross sensitivity [45] the current dosed NH_3_ concentration. The Cu-SSZ-13 SCR catalyst (Ø 5.66” = 14.4 cm, length 6” = 15.2 cm) is placed in the middle of the 40 cm resonance cavity with one RF antenna up- and one downstream of the catalyst. The ideal cylindrical cavity shape is defined by two coarse metal screens. Two thermocouples outside of the resonance cavity determine the current catalyst temperature. The last NO_x_ sensor downstream of the SCR catalyst detects the end-of-pipe emissions. Since the sensor is sensitive to both NO_x_ and NH_3_, its signal is required to be interpreted carefully. The two RF antennas are connected to the vector network analyzer (VNA, Anritsu MS46322A, RF acquisition rate: 1 Hz) by two 50 Ω coaxial cables (not shown in Figure 1). This work uses the lowest appearing resonance, the TE_111_ mode with one electrical field maximum in the cavity center. Figure 2a shows the simulated (COMSOL Multiphysics 5.1) electrical field strength (high: light, low: black) and Figure 2b the magnetic field vector of the TE_111_ mode. It is clearly visible that the SCR catalyst is located in a region with high electric field strength, since the sensitivity to permittivity changes is depending on the latter. Example transmission spectra (|*S*_21_|) with the resonance peak of the TE_111_ mode are displayed schematically in Figure 2c for the NH_3_ free state (state 1 in black) and the NH_3_ loaded state (state 2 in red). The shift of the resonance to lower frequencies, the decrease of peak height and the peak broadening due to NH_3_ storage is clearly visible.

The DEF dosing on the engine setup can be applied manually or automatically controlled on the current RF signal with defined control borders as already demonstrated in [38]. Within this study, the engine is operated at several stationary operation points with SCR catalyst temperatures between 250 and 400 °C and NO_x_ raw emissions of 100 up to 1300 ppm. Due to a compared low catalyst volume, all experiments were performed at very high space velocities (*SV*) between 90,000 and 150,000 h^−1^ that force the catalyst to operate at deliberately difficult conditions. Additionally, one operation point with continuously changing exhaust gas recirculation (EGR) rates was chosen, resulting in continuously fluctuating NO_x_ concentrations, space velocities and exhaust gas humidities. The latter was also under further investigation by analyzing the cold and warm start water influence to the RF signal.

## 4. Results and Discussion

### 4.1. RF Response Validation and Procedure for NH_3_ Storage Influence Investigations

**Figure 3:**
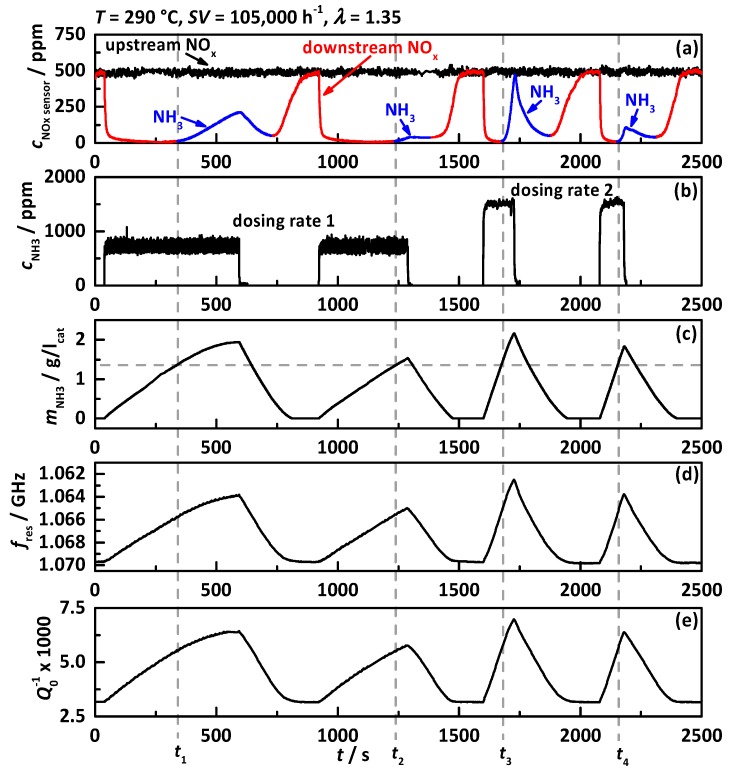
Experiment at 290 °C with *SV* = 105,000 h^−1^ and *λ* = 1.35 and two DEF dosing rates: (**a**) NO_x_ sensor signal upstream of DEF dosing (black) and downstream of SCR catalyst (red: assigned to downstream NO_x_, blue: assigned to downstream NH_3_); (**b**) dosed NH_3_ concentration determined by NO_x_ sensor signals up- and downstream of DEF dosing; (**c**) calculated amount of NH_3_ stored on the catalyst; (**d**) resonance frequency *f*_res_ in reverse scale and (**e**) the reciprocal unloaded quality factor *Q*_0_^−1^. The dashed lines indicate the loading level when first NH_3_ slip occurs.

In [38] the functionality of the RF system on the engine test bench with an iron exchanged zeolite catalyst was proven for the first time. The first experiment (Figure 3) of this paper was performed to show the same functionality for the observed Cu-SSZ-13 catalyst with a space velocity of 105,000 h^−1^, an air-to-fuel ratio of *λ* = 1.35 and a catalyst temperature of 290 °C, with (a) the signals of the NO_x_ sensors located upstream of the DEF dosing (black) and downstream of the SCR catalyst (red: assigned to downstream NO_x_, blue: assigned to downstream NH_3_); (b) the dosed NH_3_ concentration determined by the two NO_x_ sensors up- and downstream of the DEF dosing; (c) the calculated stored NH_3_ mass in gram per liter catalyst volume; (d) the resonance frequency *f*_res_ in reverse scale and (e) the reciprocal unloaded quality factor *Q*_0_^−1^.

**Figure 4:**
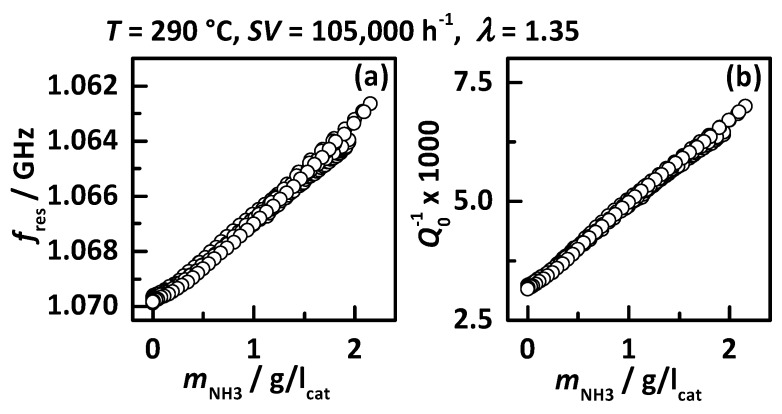
RF signal of experiment of Figure 3 at 290 °C with *SV* = 105,000 h^−1^ and *λ* = 1.35: (**a**) the resonance frequency *f*_res_ in reverse scale and (**b**) the reciprocal unloaded quality factor *Q*_0_^−1^ as a function of the calculated stored NH_3_ on the catalyst.

Within this experiment, the catalyst was loaded with NH_3_ for four times with two different DEF dosing rates, whereas dosing rate 2 injects double the amount of urea as dosing rate 1. When continuous urea dosing is applied, the downstream NO_x_ concentration drops instantaneously and shows after a short time full NO_x_ conversion. When the NH_3_ storage capacity is exceeded, NH_3_ breakthrough appears (highlighted in blue) visible in the increase in the downstream NO_x_ sensor signal (*t*_1_, *t*_2_, *t*_3_ and *t*_4_). When the DEF dosing is turned off again, the NH_3_ breakthrough decreases slowly, followed by another increase in NO_x_ sensor signal up to the upstream concentration, indicating that the catalyst is NH_3_-free again. The calculated NH_3_ mass on the catalyst shows that the critical NH_3_ storage degree appears to be around 1.4 g/l_cat_, since in all four dosing experiments NH_3_ slip is visible when this storage degree is exceeded. This proves the good reproducibility of the NH_3_ storage experiment and the chemical behavior of the catalyst. By comparing the calculated NH_3_ mass with *f*_res_ and *Q*_0_^−1^, the good correlation between both RF signals and the catalyst NH_3_ loading state is proven for Cu-SSZ-13. This relation is better visible in Figure 4 with (a) *f*_res_ and (b) *Q*_0_^−1^ as a function of stored NH_3_ mass. The linear relationship between both RF signals and the catalyst NH_3_ storage degree with no influence whether the catalyst is storing, depleting and converting NO_x_ is clearly visible as already reported in [38].

**Figure 5:**
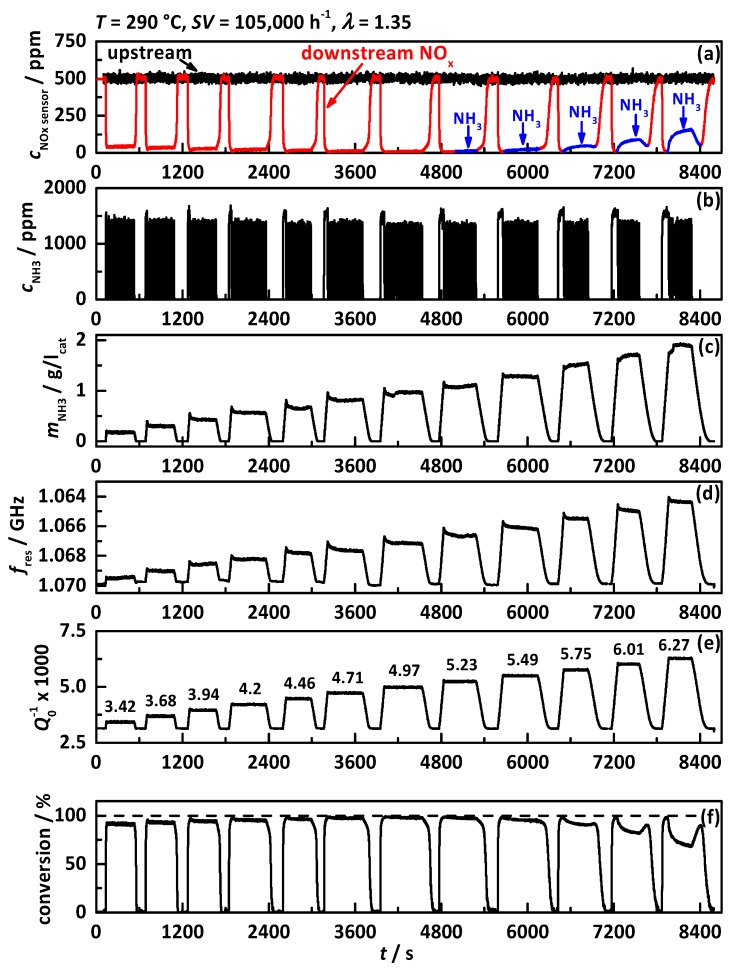
Experiment to investigate the NH_3_ storage influence to catalyst performance at 290 °C with *SV* = 105,000 h^−1^ and *λ* = 1.35: (**a**) NOx sensor signal upstream of DEF dosing (black) and downstream of SCR catalyst (red: assigned to downstream NO_x_, blue: assigned to downstream NH_3_); (**b**) dosed NH_3_ concentration determined by NO_x_ sensor signals up- and downstream of DEF dosing; (**c**) calculated amount of NH_3_ stored on the catalyst; (**d**) resonance frequency *f*_res_ in reverse scale; (**e**) the reciprocal unloaded quality factor *Q*_0_^−1^ and (**f**) the apparent NO_x_ conversion based on the sensor signals of (**a**).

With the proven functionality of the RF signals for NH_3_ storage determination, the RF signal was used for automatic urea dosing control to investigate the influence of the NH_3_ storage degree to the catalyst performance. Figure 5 shows an example for an experiment performed with a space velocity of 105,000 h^−1^, *λ* = 1.35 and a catalyst temperature of 290 °C. It displays the same signals as Figure 3 with the additional plot (*f*) of the apparent NO_x_ conversion rate based on the signals of NO_x_ sensors of (a). Within this experiment, the urea dosing was controlled to constant storage degrees by *Q*_0_^−1^. These experiments were also conducted with control on *f*_res_, leading to the same results. Starting with a low NH_3_ storage, the latter was increased stepwise, always starting with an empty catalyst. The lowest observed storage of 0.2 g/l_cat_ (corresponding to *Q*_0_^−1^ × 1000 = 3.42) already shows a high NO_x_ conversion of over 90%. By stepwise increasing the storage value, the NO_x_ conversion efficiency also increases and reaches constant full conversion at a NH_3_ storage level of 1.0 g/l_cat_ (*Q*_0_^−1^ × 1000 = 4.97). When the control value for NH_3_ storage further increases, the NO_x_ sensor downstream of the catalyst shows a slow signal increase indicating slow NH_3_ slip. This might be explained by slowly migrating NH_3_ from the front of the catalyst to its end if one constant storage value is kept for longer time. This effect gets stronger with further growing NH_3_ storage degree, until at 1.9 g/l_cat_ (*Q*_0_^−1^ × 1000 = 6.27) the downstream NO_x_ sensor shows almost 200 ppm NH_3_ signal. This experiment demonstrates that with a precise knowledge of the current NH_3_ storage the catalyst can be operated in a state with its maximum conversion efficiency and without crossing the critical storage limit for NH_3_ slip. At the observed temperature of 290 °C with a space velocity of 105,000 h^−1^, the NH_3_ storage degree of 1.0 g/l_cat_ appears to be the ideal operation point.

### 4.2. Temperature Dependency of NH_3_ Storage, NO_x_ Conversion and RF Signals

**Figure 6:**
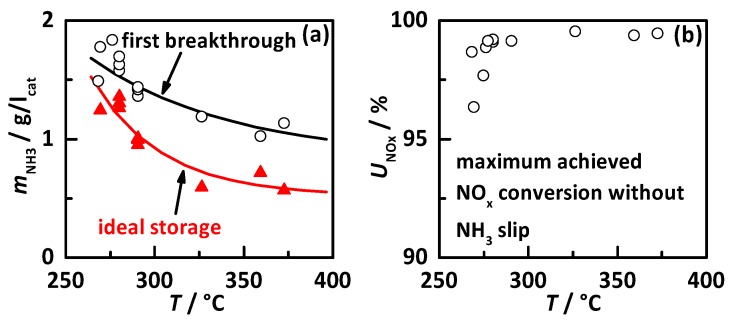
NH_3_ storage behavior of Cu-SSZ-13 with (**a**) the ideal storage degree (lowest storage for maximum conversion, red triangles) and the storage at first breakthrough at continuous urea dosing with a previously NH_3_ free catalyst (black circles) and (**b**) the maximum achieved NO_x_ conversion as a function of catalyst temperature.

The experiment discussed above was performed at various operation points in the temperature range of 250 to 400 °C. Within this study, the ideal NH_3_ storage value, i.e., the lowest NH_3_ storage when maximum NO_x_ conversion was achieved, was determined for all observed temperatures. Additionally, the storage value of first NH_3_ breakthrough was analyzed, which represents the first NH_3_ slip when the previously NH_3_ free catalyst is loaded with a constant urea dosing rate (as in the experiment displayed in Figure 3). Figure 6 shows: (a) the ideal NH_3_ storage degree (red triangles) and the NH_3_ breakthrough loading (black circles); and (b) the maximum achieved NO_x_ conversion without NH_3_ slip. Both storage values are also fitted with an exponential decay function (solid line). One can see that both storage curves decrease with increasing catalyst temperature, since the NH_3_ desorption is thermally activated. This temperature dependence fits well to the expected behavior relying on results of previous work on the gas test bench [37,40] and to current control models [11]. Both curves are close together at the lower observed temperatures, whereas the ideal storage curve shows a stronger decay with temperature than the first breakthrough. This might be related to the better reaction kinetics at higher temperatures that does not require high NH_3_ surface coverage. The achieved NO_x_ conversion at stationary operation points was always higher than 95% and increases at temperatures above 280 °C to 98%, due to the thermally activated reaction kinetics. The best value of 98% may also be related to the accuracy limit of the used NO_x_ sensors and might represent full conversion, even at the observed forcing conditions with extreme high space velocities. It should be noted here, that the catalyst was operated at very unusual high space velocities. At typical space velocities, an even better performance can be expected.

**Figure 7:**
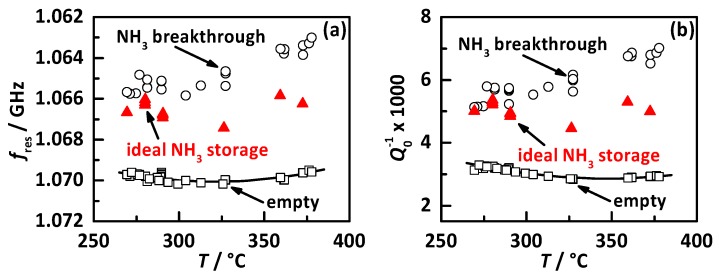
RF signals as function of catalyst temperature for the empty state (black squares), the NH_3_ breakthrough (black circles) and the ideal NH_3_ storage degree (red triangles): (**a**) the resonance frequency *f*_res_ in reverse scale and (**b**) the reciprocal quality factor *Q*_0_^−1^.

The determined temperature dependency of the RF signals is displayed in Figure 7 for the NH_3_ free state (black squares), the NH_3_ breakthrough loading (black circles) and the ideal NH_3_ storage degree (red triangles), with (a) *f*_res_ in reverse scale and (b) *Q*_0_^−1^. In principle, it obvious that both RF signals appear to have a very similar temperature dependent behavior. Without NH_3_, they show in the lower temperature region with increasing temperature a decrease in the opposite direction of the signal as it corresponds to NH_3_ loading. For higher temperatures, a small increase in direction of the NH_3_ signal is visible. This behavior might be explained by several reasons related to material effects and the resonator cavity geometry. At the lower temperatures, the zeolite catalyst stores H_2_O at the same storage sites and with a similar effect to the RF signal as NH_3_. With increasing temperature, the ability to adsorb H_2_O decreases and so do both RF signals. At the higher temperature regions, H_2_O has almost no influence, but instead, the charge carriers inside the zeolite structure get more mobile, which also leads to an increase in RF signal. But this effect is comparably small to H_2_O, which can be seen for *Q*_0_^−1^. The resonance frequency is additionally affected, since the resonator cavity expands with temperature. This geometry increase leads to a proportional decrease in resonance frequency and explains the behavior of *f*_res_ at the higher temperature for the empty state. Since *Q*_0_^−1^ (the dielectric losses) are not affected by the geometry, *Q*_0_^−1^ shows a smaller temperature dependency. The NH_3_ breakthrough curve shows an increase of signal intensity for *f*_res_ and *Q*_0_^−1^ with increasing temperature, whereas the stored NH_3_ mass decreases. The ideal NH_3_ storage curves are in the lower temperature region close to the breakthrough values and move towards higher temperatures roughly into the middle between the empty and breakthrough values, as already seen in Figure 6.

**Figure 8:**
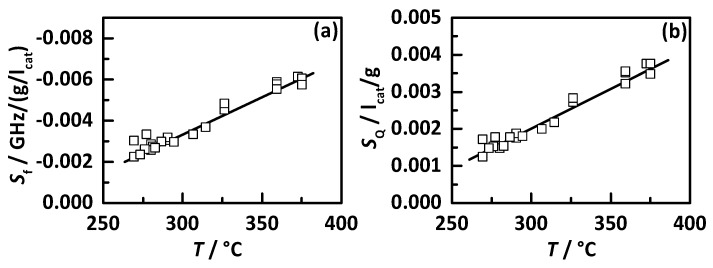
Sensitivity of the RF signals to NH_3_ storage with (**a**) *S*_f_ of the resonance frequency *f*_res_ and (**b**) *S*_Q_ of the reciprocal quality factor *Q*_0_^−1^ as a function of catalyst temperature.

The experimental results shown in Figure 4 already proved the linear response of both RF signals to NH_3_ storage and this behavior was also seen in all other experiments in the whole temperature range. Therefore, the following discussion focusses on the sensitivity of both RF signals to NH_3_ storage, i.e., the slopes assuming an ideal linear relationship. Figure 8 displays the sensitivities to NH_3_ storage (a) *S*_f_ for *f*_res_ and (b) *S*_Q_ for *Q*_0_^−1^ as a function of catalyst temperature. It is clearly visible that the sensitivities of both RF signal increase in an almost linear manner with temperature. This might also be caused by the higher mobility of charge carriers at higher temperatures and explains the increase in NH_3_ signal intensity in Figure 7 while the stored NH_3_ mass decreases (see Figure 6). Similar effects have already been reported for metal exchanged zeolites in previous work [37,40].

The results of this study now offer the chance for a temperature dependent calibration of the RF system for the observed catalyst Cu-SSZ-13 to monitor and control the current NH_3_ storage under transient conditions. Therefore, the quadratic fitting functions for the empty state (shown in Figure 7) and the linear fitting functions for the sensitivities (shown in Figure 8) can be used for real time NH_3_ storage determination and is in focus of forthcoming work.

### 4.3. Influences of Humidity Changes and Cold Start Water

**Figure 9:**
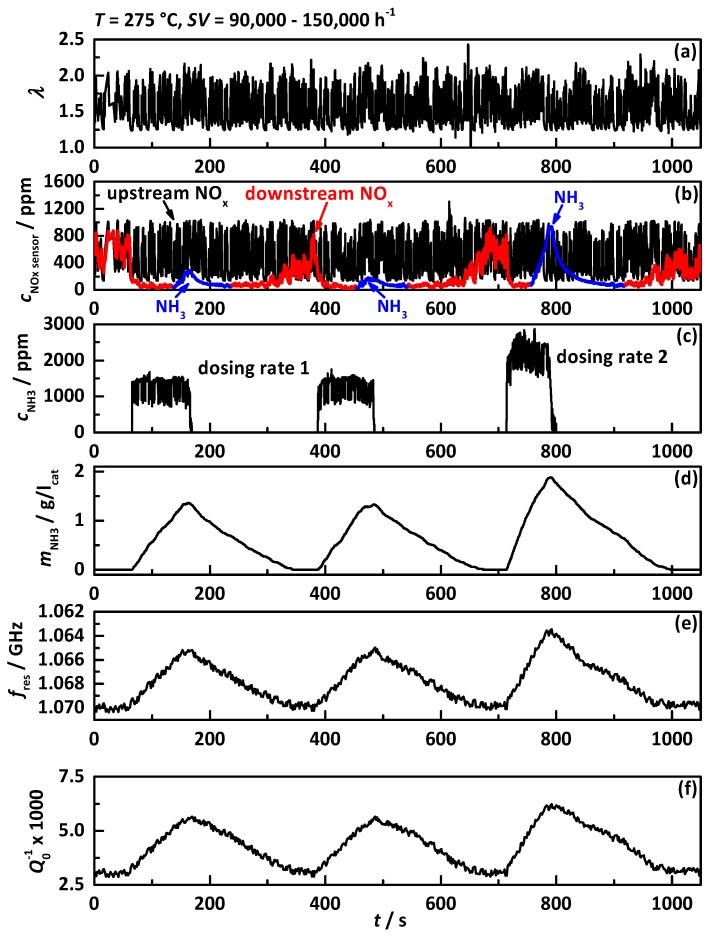
Experiment with continuously changing EGR rate with a catalyst temperature of 275 °C and a space velocity between 90,000 and 105,000 h^−1^ with (**a**) the *λ* signal of the upstream NO_x_ sensor; (**b**) the NO_x_ sensor signals upstream of the DEF dosing (black) and downstream of the SCR catalyst (red: downstream NO_x_, blue: downstream NH_3_); (**c**) dosed NH_3_ concentration determined by NO_x_ sensor signals up- and downstream of DEF dosing; (**d**) calculated amount of NH_3_ stored on the catalyst; (**e**) resonance frequency *f*_res_ in reverse scale and (**f**) the reciprocal unloaded quality factor *Q*_0_^−1^.

The experiments in [38] already showed the influence of humidity in the exhaust gas to the RF signal for an iron exchanged zeolite, leading to a decrease in signal accuracy. The identical experiment with a continuously changing EGR rate that causes varying *λ* values, varying space velocities, as well as varying raw NO_x_ emissions at a constant catalyst temperature has been repeated for the more recent serial catalyst Cu-SSZ-13 and is displayed in Figure 9. The *λ* signal of the upstream NO_x_ sensor in (a) shows the continuously changing EGR rate and takes values between 1.25 and 2.25. Within this experiment, the catalyst is loaded with NH_3_ three times with two different urea dosing rates, each time until NH_3_ breakthrough is detected by the downstream NO_x_ sensor (highlighted in blue). The signals of the NO_x_ sensors upstream of the DEF dosing (black) and downstream (red) of the catalyst in (b) show without dosing the identical noisy behavior mirroring the *λ* signal. With applied urea dosing the downstream NO_x_ sensor signal drops instantaneously and shows high but no full conversion until the NH_3_ breakthrough appears. The fact that the catalyst is not able to achieve full conversion and the downstream NO_x_ sensor still detects roughly 50 ppm might be explained by the high space velocity (or low catalyst volume) and the short-term high NO_x_ concentration up to 1000 ppm. The dosed NH_3_ concentration in (c), calculated from the NO_x_ sensor signal up- and downstream of the DEF dosing, switches between two concentrations since the exhaust gas mass flow changes continuously but the dosing rate remains constant. Both RF signals *f*_res_ in (e) and *Q*_0_^−1^ in (f) still correlate very well to the calculated stored NH_3_ mass in (d). However, both RF signals appear more affected by the changing humidity for Cu-SSZ-13 compared to the iron exchanged zeolite from [38], resulting in a bigger uncertainty, as can be learned from the more “noisy” curves in (e) and (f).

**Figure 10:**
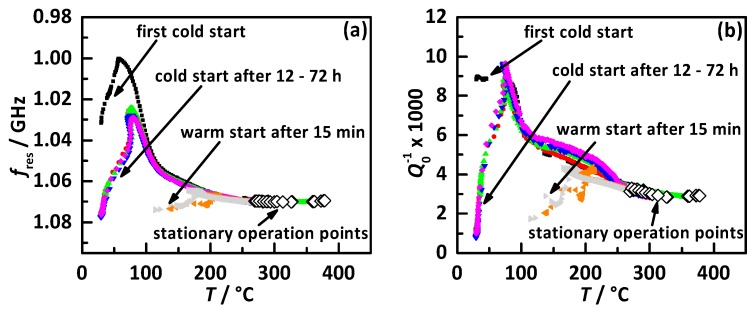
RF signals as a function of catalyst temperature for cold and warm starts under different start conditions (colored symbols) and under stationary conditions (white diamonds from Figure 7) for (**a**) *f*_res_ and (**b**) *Q*_0_^−1^.

Besides small humidity changes under transient conditions, a much bigger effect might be caused by adsorbed water as it appears at cold starts. This has already been observed for a TWC-coated GPF in [30]. Cold start water adsorption is also under investigation to better understand zeolite SCR catalysts and to improve the conversion efficiency especially for continuously decreasing exhaust gas temperatures [46]. Therefore, the start behavior of the RF-SCR system has been analyzed under different start conditions. The results are displayed in Figure 10 with (a) *f*_res_ in reverse scale and (b) *Q*_0_^−1^ as a function of temperature. The start procedure and the ambient temperature were identical for each run. Each performed cold or warm start is marked by a different color. Additionally, the values of the stationary operation point with NH_3_ from Figure 7 are added (white diamonds). The cold starts were conducted with a catalyst start temperature of 25 °C, the warm starts of 120 °C. The basic curve of both RF signals for a cold start is as follows. First, the signals shift into the same direction as NH_3_ storage would cause until they reach their maximum roughly around 75 °C. With further increasing temperature, they shift back in the opposite direction until they are identical to the values of the stationary operation points above temperatures of 250 °C. The very first cold start begins with values of *f*_res_ = 1.01 GHz and *Q*_0_^−1^ × 1000 = 9.0 and shows for *f*_res_ the biggest shift. All following cold starts started at *f*_res_ = 1.08 GHz and *Q*_0_^−1^ × 1000 = 1.0 with no influence whether the engine was off for 12 or 72 h. Each cold start showed above 100 °C an almost identical behavior and this proved the high reproducibility of the cold start influence of RF-SCR system. A possible explanation for the differing first cold start might be the fact that the catalyst has not been heated before and it was exposed to room humidity for a long time. Between the different cold starts, the catalyst was not able to adsorb the same amount of water than before. The performed warm starts fit after a short time after the engine started, roughly above temperatures of 180 °C very well to the cold start curves.

The frequency shift of the first cold start related to the stationary operation point of 300 °C was ca. 70 MHz. For *Q*_0_^−1^ × 1000 the same shift was around 6.5. The maximum signal shift related to NH_3_ at 300 °C was for *f*_res_ ca. 5 MHz and for *Q*_0_^−1^ × 1000 ca. 2.8 (see Figure 7). The observed maximum effect related to cold start water was for *f*_res_ 14-times and for *Q*_0_^−1^ three times higher than the maximum NH_3_ response. This demonstrates that the resonance frequency (*f*_res_) is much more affected by water compared to the loss-related value of *Q*_0_^−1^. A possible explanation for this effect might be that *f*_res_ is mostly affected by polarization effects (please note the high dipole moment of gaseous H_2_O of 1.84 D [47]) compared to *Q*_0_^−1^, which represents the dielectric and conductivity losses. The polar nature of the water molecule might cause this big difference. Nevertheless, even when cold start water has the demonstrated huge effect to the RF signals, this effect happens only at much lower temperatures than the SCR typically is operated. When the catalyst has reached its usual operation conditions, no more cold start water is stored on the catalyst and the RF catalyst monitoring is not affected.

## 5. Conclusions and Outlook

For several years, RF-based catalyst state monitoring has been a focus of research and development as the only direct measure of the current NH_3_ storage on SCR catalysts. Whereas most studies were performed with synthetic exhaust on the laboratory scale using gas test benches, the big step towards application size and real exhaust measurements on an engine dynamometer setup was achieved recently and proved the ability of the RF technique under stationary conditions [38]. It could be demonstrated that a direct urea dosing control on the NH_3_ storage degree determined by the RF signal is possible. Furthermore, this technique allows precise investigations of the NH_3_ storage influence to the catalyst performance and NH_3_ slip. This paper continues this work with focus on the temperature dependency of the RF signal and the NH_3_ storage behavior. Additionally, the influence of exhaust gas humidity and especially of cold start water was investigated.

The observed sample was a well-studied commercial and serial type Cu-exchanged zeolite-based SCR catalyst (Cu-SSZ-13), in contrast to [38], where a mostly unknown serial-type iron containing zeolite-based SCR catalyst was used. The catalyst volume was increased compared to the sample of [38], but still smaller than in common application size and forced the catalyst to operate at very high space velocities. As a first step, the full functionality of the RF system was demonstrated and the linear correlation of both RF signals *f*_res_ and *Q*_0_^−1^ and the current NH_3_ storage was proven for Cu-SSZ-13. Subsequently, the RF signal was used to investigate the NH_3_ storage influence to the catalyst performance with respect to NO_x_ conversion and possible NH_3_ slip in a temperature range from 250 to 400 °C. Based on these experiments, an ideal NH_3_ storage curve as a function of catalyst temperature was developed and showed the same basic behavior as in established control approaches [11]. Furthermore, a full temperature dependent calibration map with fitting functions for the NH_3_-free state and the sensitivity of both RF signals to NH_3_ storage was created. It is noteworthy that the sensitivities of *f*_res_ and *Q*_0_^−1^ showed an almost linear increase with temperature. With this calibration functions, a temperature independent NH_3_ storage determination seems possible and is in focus of the upcoming work.

The humidity influence on the accuracy of the RF approach already described in [38] was also confirmed for Cu-SSZ-13. The influence of cold start water was investigated much more in detail, indicating that cold start water leads to a much higher signal than NH_3_ at lower temperatures. Nevertheless, at SCR active temperatures, the cold start water has already desorbed of the catalyst and has no more impact on the RF signals. All cold and warm start experiments showed a very reproducible behavior and fit well with the results of the stationary operation point in the higher temperature region.

The upcoming work will focus on the application of the developed calibration under transient conditions for Cu-SSZ-13. Therefore, different target NH_3_ storage curves (for example the determined ideal NH_3_ storage curve) will be applied to investigate their influence to the catalyst performance under more realistic conditions. Additionally, improvements for the accuracy of the RF system to compensate humidity changes by using the known *λ* value deserves further consideration. We intend to test various current SCR catalysts systems at different catalyst aging states to predict their ability for the RF approach and their aging behavior. In addition, possible effects due to poisoning deserve to be studied. Over all these, the biggest target is still the application on the road with an RF controlled or RF assisted model-based SCR system.
